# What’s not to learn? AI meets parasitology

**DOI:** 10.1128/jcm.01451-25

**Published:** 2025-12-08

**Authors:** James E. Kirby, Ramy Arnaout

**Affiliations:** 1 Department of Pathology, Beth Israel Deaconess Medical Center 1859 , Boston, Massachusetts, USA; 2 Harvard Medical School 1811 , Boston, Massachusetts, USA; Mayo Clinic Minnesota, Rochester, Minnesota, USA

## Abstract

Although artificial intelligence—particularly large-language models—receives daily attention, the application of AI to image-recognition challenges in clinical microbiology has been under development for several years. In the accompanying article, B. A. Mathison, K. Knight, J. Potts, B. Black, et al. (J Clin Microbiol 63:e01062-25, 2025, https://doi.org/10.1128/jcm.01062-25) (in collaboration with ARUP Laboratories and TechCyte) describe a trained convolutional neural network (CNN) that reviews wet-mount parasitology smears with accuracy and analytical sensitivity exceeding that of a cohort of highly trained medical technologists. The impressive results were enabled by an extensive, globally sourced training set. These findings constitute Part II of the authors’ earlier Journal of Clinical Microbiology publication on CNN-based diagnosis of trichrome-stained smears and provide a robust proof-of-concept for integrating AI into clinical microbiology workflows. We comment on the translatability of this technology to routine clinical laboratories.

## COMMENTARY

Stool parasitology exemplifies a highly complex clinical laboratory diagnostic task. Wet-mount preparations contain a wide variety of eggs, protozoal trophozoites, cysts, and helminth forms that must be distinguished. Subtle morphological differences between pathogenic and non-pathogenic forms challenge even experienced diagnosticians. Rare pathogens may be seen only annually, once per decade, or never in smaller laboratories, limiting diagnostic competence. Even a single egg or protozoan can signify disease. Consequently, any lapse in attention or incomplete slide scanning can produce false-negative results. A parallel exists in surgical pathology, where pathologists search for micrometastases; however, tissue architecture provides contextual cues that aid detection. In contrast, parasites appear against an amorphous background of bacteria, yeast, and food debris, making the task daunting. Experience remains critical; however, the technologist workforce is increasingly stressed and younger, at times with limited experience. A readily available “helping eye” that encapsulates years of parasitology expertise would be valuable.

Convolutional neural network (CNN)-based AI, which excels at image recognition, has long been viewed as a potential supplement to parasitology and other image-intensive tasks in clinical microbiology. However, asserting that a CNN trained to recognize dogs and cats will automatically recognize *Acanthamoeba* is insufficient; realizing this capability requires dedicated development. In this regard, the study by Matheson et al. robustly bridges theory and practical implementation. Broadly, the authors trained a CNN to identify 27 stool parasites in wet-mount preparations. To enhance generalizability, they incorporated globally sourced samples preserved in commonly used fixatives and imaged on multiple modern slide scanners. Collecting such a diverse, worldwide data set was a Herculean effort that supplied the model with sufficient data to achieve excellent performance.

The primary workflow challenge in clinical parasitology is screening large numbers of samples for rare eggs, worms, trophozoites, or cysts amid abundant commensal flora and stool debris. To quantify the confidence of the AI tool’s predictions, scanner-specific cutoff thresholds were derived using precision-recall curves. Precision corresponds to positive predictive value, and recall corresponds to sensitivity. Precision-recall curves are especially useful for evaluating rare-event detection because they emphasize performance on the positive class. Accordingly, the authors adjusted the AI decision thresholds to maximize recall (sensitivity) while tolerating a modest reduction in precision (positive predictive value). As expected, the models achieved the highest performance when both training and deployment used images from a single slide-scanner type.

The AI’s analytical sensitivity matched or exceeded that of several technologists, and during validation, it identified pathogens in many specimens that had been reported negative by conventional microbiology. The technology is therefore presented as an untiring screening system that correctly classifies the vast majority of cases, with occasional errors mainly in distinguishing non-pathogenic protozoa and degenerated *Strongyloides* forms. Overall, the reported data indicate that the AI model provides superior accuracy and sensitivity compared with the standard of care.

The authors acknowledge limitations, including difficulty recognizing parasites for which insufficient training data exist and the risk that out-of-focus rare pathogens may be missed or misclassified. Consequently, the system is intended primarily as a screening tool. In practice, the AI forwards selected images to a technologist for final confirmation and discrimination, an approach previously described as “Technologist Assist” (1, 2). Misclassifications (wrong IDs) can thus be corrected by the technologist. Rather than spending hours examining uninformative stool material, technologists can focus on a limited set of high-yield images that likely contain pathogens. The model detected markedly more pathogens and achieved higher resolved accuracy than conventional parasitology workflows.

A logical next question is how this methodology compares with alternative approaches. Diagnostic parasitology differs from other image-based clinical diagnostics, such as chest X-ray, CT, cardiac MRI, or surgical pathology interpretation, where AI models can also assist in diagnosis. In radiology, for example, image interpretation is typically integrated with clinical information to provide a diagnosis (3, 4). However, in parasitology, diagnosis can be rendered through either highly operator-dependent or model-dependent morphological assessment alone or alternatively through the detection of molecular signatures (e.g., nucleic acids, antigens) that provide highly specific diagnostic information. The latter are usually performed using largely operator-independent clinical chemistry or molecular microbiology technology that, instead of cumulative training and experience, relies on robust assay design and application of rigorous quality control and good laboratory practice. The availability of potentially compelling alternatives raises the question of whether AI-assisted parasitology will achieve widespread adoption relative to competing technologies. More broadly, we must define the optimal role of AI and machine learning in diagnostic microbiology to maximize their utility alongside existing methods.

How can the present AI model be integrated into a clinical microbiology laboratory? Validating a laboratory-developed test (LDT) of this complexity exceeds the resources of most clinical laboratories, which limits full local implementation to high-volume reference labs. The slide scanners required for the algorithm are costly, but they enable remote image analysis. Therefore, two deployment pathways are possible: (i) an AI model validated elsewhere and distributed to sites as a Food and Drug Administration (FDA)-cleared system, based on the high bar for local LDT development, or (ii) a service accessed via subscription or collaboration, wherein wet-prep and trichrome-stained slides are prepared and scanned locally for centralized remote analysis. The centralized model is attractive because a broad user network can continuously generate new training data and iteratively improve the model.

Surgical pathology is moving toward full digitization of histology slides, allowing conventional microscopy-level review and the addition of AI/ML-based diagnostic analysis. Whole-slide scanners comparable with those described in the manuscript typically cost around $100,000. Existing anatomic-pathology scanners may be repurposed for parasitology if they fit workflow requirements and can accommodate varied scanning parameters, thereby reducing the cost of patient-reportable results. Alternatively, lower-cost microscopes equipped with automated stages and autofocus could be dedicated to parasitology scanning to provide an additional way to access a validated AI model.

From a regulatory perspective, local slide scanning followed by image transmission to a central reference site raises specific validation and compliance questions. Local sites must verify that their scanners reproduce image quality comparable to the validated central workflow and conduct site-specific verification studies. Image transfer must comply with Health Insurance Portability and Accountability Act (HIPAA) of 1996 security rules, requiring encrypted transmission and controlled access for local review. Alternatively, specimens can be sent to a reference laboratory that already holds a validated AI LDT, although this will increase turnaround time.

Compared with multiplex molecular panels that potentially could detect a similar array of pathogens, this AI-based imaging technology offers distinct advantages and limitations. Molecular diagnostics automate testing, eliminating technologist-dependent specimen concentration, wet-prep preparation, trichrome staining, and the need for pathogen-specific specialty acid-fast and chromotrope stains. Large commercial respiratory and blood-culture identification panels already exist that can provide the type of broad multiplex detection desired. Therefore, molecular diagnostics could theoretically replace or supplement microscopy-based screening for large pathogen sets and could be developed using *in silico* analysis and synthetic targets to avoid the necessity of acquiring specimens with rarely detected analytes during validation. Such assays can be engineered to achieve very high specificity and sensitivity and process high specimen volumes. They could also address several limitations of image-based categorization. For example, microscopy (and therefore AI trained on microscopy images) cannot reliably distinguish *Entamoeba dispar* from *E. histolytica*.

However, there is always a balance between diagnostic accuracy, sensitivity, and cost. Scaling, for example, may also favor image-based analysis. Real-world market data indicate that assembling a comparably large multiplex molecular panel is difficult. No commercially available FDA-cleared panels approach the pathogen breadth examined in this study; most detect only two to four common stool parasites, with a single panel covering eight. Limited commercial incentive and potentially technical issues appear to hinder both assay-kit development and LDT-based efforts to target rarer intestinal pathogens that are infrequent in high-income countries. The peer-reviewed literature on highly multiplexed stool-parasite panels, especially those matching the sensitivity of conventional ova-and-parasite microscopy across many organisms, is scant. This creates a niche for advanced AI-driven parasite detection to complement molecular or antigen tests for the most common pathogens. The establishment of the broad pathogen-detection panel in this work can also serve as a stand-alone, comprehensive examination.

Will AI-based image analysis become mainstream in clinical microbiology? The field comprises tasks of varying complexity, each with distinct specificity and enumeration requirements. The simplest application is the binary interpretation of auramine-rhodamine fluorescence for acid-fast bacilli. This “yes/no” fluorescence readout, based on a limited set of morphologies, is already commercialized. More advanced tasks include automated Gram-stain interpretation, which must assess multiple morphologies and staining characteristics in positive blood cultures and identify epithelial and white-blood cells in primary specimens (1, 2). Integrating these analyses with automated microscopy and colony-growth recognition could support total-laboratory automation. Such capabilities are expected to become routine in future microbiology laboratories, analogous to the adoption of AI/ML for hematology smear review. Even more complex AI image-recognition tasks—such as distinguishing a wide range of fungal-mold morphologies or comprehensive parasitology screening—are likely to yield substantial efficiency gains for high-volume specialty and reference laboratories.
